# Implementing a successful patient navigation program for follow-up colonoscopy: Lessons from the PRECISE study

**DOI:** 10.1371/journal.pone.0343659

**Published:** 2026-03-18

**Authors:** Jamie H. Thompson, Jennifer L. Schneider, Jennifer S. Rivelli, Priyanka Gautom, Jeffery Gibbs, Neha Yadav, Ricardo Jimenez, Gloria D. Coronado

**Affiliations:** 1 Kaiser Permanente Center for Health Research, Portland, Oregon, United States of America; 2 Sea Mar Community Health Centers, Seattle, Washington, United States of America; 3 University of Arizona Cancer Center, Tucson, Arizona, United States of America; 4 Mel and Enid Zuckerman College of Public Health, University of Arizona, Tucson, Arizona, United States of America; Virginia Mason Franciscan Health, UNITED STATES OF AMERICA

## Abstract

**Background:**

Colorectal cancer (CRC) screening using annual fecal immunochemical tests (FIT) is an effective strategy to reduce CRC incidence and mortality. However, nearly 50% of patients with abnormal FIT results fail to complete the necessary follow-up colonoscopy. Patient navigation (PN) can provide crucial support for colonoscopy completion. Despite PN’s effectiveness, there is limited knowledge about its optimal implementation in community health settings. This manuscript presents lessons learned from a PN intervention, including successes, challenges, and recommendations, which are essential for improving future PN programs in community health centers.

**Methods:**

The PRECISE study is a patient-randomized trial of PN vs usual care for follow-up colonoscopy at a Federally Qualified Health Center in Washington state. The comprehensive implementation support for the patient navigation intervention included trainings, technical assistance, and quality assurance session to equip navigators with the skills needed to effectively guide patients to colonoscopy completion. Study staff tracked implementation support adaptations and lessons learned using data sources including PN training program reflections and patient navigator debrief interviews**.** We examined successes, challenges, and recommendations in five main categories: 1) patient navigation delivery, 2) implementation support and quality assurance, 3) staffing, 4) partnerships and resources, and 5) data tracking.

**Results:**

Navigators found the training sufficient to successfully implement the program, but pointed to challenges related to workloads, documentation, and partnerships with gastroenterology practices. Key lessons learned included streamlining patient navigation topics to enhance outreach, maintaining effective implementation support and quality assurance elements, ensuring adequate staffing, fostering partnerships with GI practices, and implementing unified data tracking systems.

**Conclusion:**

Our findings can guide future efforts to implement and sustain patient navigation programs for follow-up colonoscopy after abnormal FIT testing in community-based settings.

## Introduction

Colorectal cancer (CRC) is a leading cause of cancer deaths in the United States, and routine screening can reduce CRC incidence and mortality by finding and removing precancerous growths or cancer at early treatable stages [1]. Annual fecal immunochemical testing (FIT) is a US Preventive Services Task Force-recommended CRC screening modality, and is an important component of strategies for meeting CRC screening targets [2–7]. However, the benefits of FIT testing can only be achieved if individuals with abnormal test results obtain a follow-up colonoscopy. Delaying the receipt of a follow-up colonoscopy by six months or more is associated with higher rates of advanced stage detection and death from CRC [8,9].

Patient navigation (PN) programs are designed “to promote access to timely diagnosis and treatment of diseases by eliminating barriers to care [10]” by providing patients with a trained navigator who helps them through the stages of the screening process, including interpreting results and making and preparing for appointments. A recent evaluation of a New Hampshire-based PN program to raise colonoscopy rates reported dramatic improvements in six-month receipt of colonoscopy (96% for navigated patients vs. 69% for non-navigated patients) [11]. Moreover, the program led to a near-elimination of appointment no-shows or cancellations and better documentation of colonoscopy procedures in clinic medical records. PN programs have also been shown to improve patient satisfaction and reduce health disparities and health care costs [12].

A hallmark of PN programs is that they are delivered in a flexible manner to meet patient needs for emotional, practical, and logistical support. Integrating PN into cancer prevention and early detection efforts has been effective, but few publications speak to how programs or their implementation are adapted for specific populations or settings [13]. Such data is needed to allow for flexible and successful implementation of the PN model across a wide variety of health care settings.

We conducted a large individual randomized clinical trial, the Predicting and Addressing Colonoscopy Non-adherence in Community Settings (PRECISE) to assess the effectiveness and implementation of an adapted version of the New Hampshire PN program for follow-up colonoscopy among patients with an abnormal fecal test result. This manuscript offers a qualitative analysis on the lessons learned from the implementation of this PN program during the COVID-19 pandemic at a Federally Qualified Health Center in Washington state. The results stem from PN training program reflections and navigator debrief interviews that describe program successes, challenges, and recommendations. These findings are crucial for informing future efforts to design and implement PN programs to better meet patient needs, particularly in community health center settings.

## Methods

The PRECISE trial is a partnership between Sea Mar Community Health Centers headquartered in Seattle, Washington and Kaiser Permanente Center for Health Research in Portland, Oregon. The overall aim was to test the effectiveness of a PN intervention to improve follow-up colonoscopy completion in a community health center setting. The PRECISE study was approved by the Kaiser Permanente Northwest (KPNW) Institutional Review Board (IRB) (Protocol number: STUDY00000779), with ceding agreements from relevant institutions. The need for informed consent was waived due to minimal risks to patients. The study is registered on ClinicalTrials.gov (NCT03925883) and the full study protocol has been published elsewhere [14]. References to the trial registration and protocol are included to provide context for the originating study from which these qualitative insights were drawn.

### Setting

Sea Mar Community Health Centers (Sea Mar) is a federally qualified health center (FQHC) that operates 34 clinics, serves over 250,000 patients in Washington State, and has a patient population that is 37% Latino/Hispanic. As part of usual care, Sea Mar operates a mailed FIT outreach program that mails FIT kits (OC-Auto-FIT, Polymedco, Cortlandt Manor, NY) and sends automated reminders to eligible patients. Sea Mar patients with an abnormal FIT result are referred to one of over 70 outside gastroenterology (GI) facilities for colonoscopy in western Washington or northern Oregon.

### Population and trial outcomes

The PRECISE study enrolled 985 patients, with 489 randomized to the PN intervention and 496 to usual care. Eligible patients were ages 50–75, had an abnormal FIT result in the past month, and were due for a follow-up colonoscopy. Patients were randomized to the intervention (PN) or a control condition (usual care). The primary outcome of the trial was colonoscopy completion within one year of an abnormal FIT result. Secondary outcomes included time to colonoscopy receipt, adequacy of bowel preparation, and differences in effectiveness by subgroups (including baseline probability of colonoscopy completion).

### Trial suspension during the early COVID-19 pandemic

PRECISE study patient identification took place between July 29, 2019, and April 4, 2022. During the initial stages of the COVID-19 pandemic (spring of 2020), colonoscopy completion was affected by changing GI practice policies due to distancing requirements and procedure capacity challenges. Practices initiated short-term care suspensions and other policies to triage access to colonoscopy. With approval from the funding organization and support from the study data safety monitor, we suspended PRECISE recruitment for five months due to COVID-19, starting March 16 and ending August 16, 2020. This was consistent with national recommendations to postpone cancer screening. Once the study resumed, we trained patient navigators to support patients on their path to completing a colonoscopy by helping them understand changing GI policies (e.g., masking, pre-procedure COVID-19 testing).

### Intervention

#### Patient navigation.

**Fig 1:**
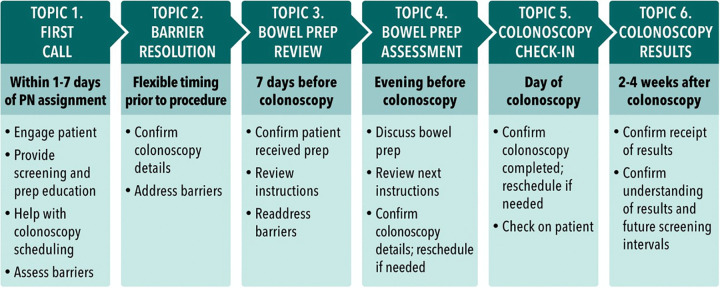
Patient navigation 6 topic protocol.

Our PN protocol was adapted from Dr. Lynn Butterly’s New Hampshire Colorectal Cancer Screening Program (NHCRCSP), a telephone-based PN program that consists of six topic areas (Fig 1)[15]. Full intervention details are reported elsewhere [14].

The clinic employed one full-time care coordinator to serve the role of the patient navigator and conduct all outreach for eligible study patients allocated to the intervention condition. Throughout the trial (August 2019 – April 2022), we trained a total of nine navigators. All navigators were bilingual in English and Spanish, worked flexible hours to accommodate patient follow-up, and had backgrounds in either basic healthcare and medical terminology or customer service.

The patient outreach process began with an introductory letter, followed by telephone calls (three cycles of six call attempts, totaling a maximum of 18 call attempts), text messages, and close-out letters if the patient declined or could not be reached, all within 30 days of mailing the introductory letter. The navigator attempted to reach patients by calling up to three times a day, but leaving only one voicemail per day, on different days of the week and at various times of the day. Initial engagement was done Monday through Friday between 8 AM and 6:30 PM. If requested by patients, navigators accommodated their schedules and called during the weekends or evening hours.

#### Implementation support and quality assurance.

To support implementation of the intervention, trial staff provided training for patient navigators as well as weekly technical assistance and quality assurance meetings. Additional implementation support ad-hoc technical assistance and booster trainings as needed.

Navigator Training. Initial navigator trainings used an intensive eight-week in-person training schedule based on the original NHCRCSP program [15]. We included instruction on colon health education (i.e., CRC screening, colonoscopy procedure, bowel preparation), barriers and effective messaging to promote colonoscopy following abnormal fecal testing, program workflow, motivational interviewing and role-playing, and data tracking.

**Fig 2:**
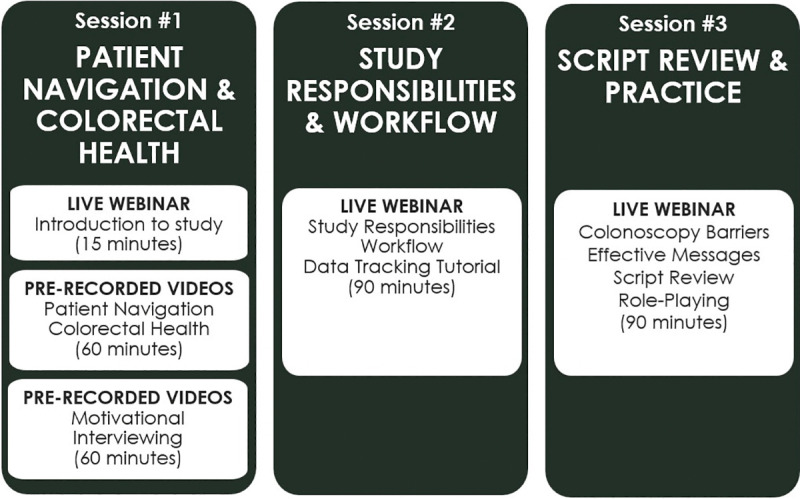
Virtual patient navigation training program overview.

During the COVID-19 pandemic, we converted to a virtual training platform to satisfy high staff turnover and COVID-19 distancing requirements (Fig 2). Based on the 8-week in-person training, we developed 11 training video modules (https://research.kpchr.org/engage/Trainings) and combined them into pre-recorded videos and live webinar sessions. Depending on trainee preference, the training was delivered over the course of several days or one long day. Content was optimized to focus specifically on three core topic areas: 1) navigation basics and colorectal health, 2) study responsibilities and workflow, and 3) script review and practice.

A short 3-minute video was also created to train navigators on how to speak with patients about the impacts of COVID-19 on colonoscopy screening. The video included changes to GI practice policies, including policies to triage access to colonoscopy procedures, conversion of in-person pre-procedure visits to telehealth, the added requirement of a negative COVID-19 test result prior to the colonoscopy, masking requirements, and restrictions on visitors in waiting and exam rooms.

The research team also created messages to reassure patients about seeking necessary care during the pandemic. Examples included: *“Getting a colonoscopy is safe right now”* and *“Your doctor’s office is prepared to care for all your needs safely.”* Other messages emphasized the steps that clinics took to protect patients, such as *“Our clinic regularly cleans and disinfects exam rooms and instruments; we limit visitors and require social distancing, we set appointment times to reduce time in waiting rooms, and we require masks.”*

Technical Assistance. Weekly technical assistance meetings occurred between the PRECISE project manager and navigator after the initial training. The weekly meetings addressed how the program was operating, case review and data management, as well as providing navigators with a chance to report on system-level barriers hindering implementation of the intervention.

Quality Assurance. Quality assurance meetings with the navigator were held weekly for 30–60 minutes and were led by a study trainer (JSR). During these sessions, the PN trainer assessed several key components: accuracy of the information provided to participants, navigator confidence, effectiveness of motivational interviewing techniques to address barriers or hesitancy to follow-up colonoscopy, and overall nature of navigator-patient interactions. Each quality assurance session was tracked using an Excel tracking tool in which the navigator recorded tasks completed during the phone call, barriers expressed by the participant, and feedback provided to navigator by the trainer.

#### Partnerships.

To increase PN success, several engagement approaches were used to help the navigator access community resources to support patients in completing a colonoscopy procedure. The research study team facilitated conversations between the patient navigator and local GI practices to assist with appointment scheduling. Relationships were also built with the Washington State Department of Health that provided reduced-cost specialty care for individuals who were uninsured or underinsured. Finally, patients were offered free rideshare services provided by a community grant from Lyft, Inc. (San Francisco, CA).

#### Data tracking system.

The data tracking system used in this program was built in REDCap (Research Electronic Data Capture), a secure web-based application designed for data collection and management in research. Data collected included navigation outcomes for each of the six topic areas, including patient barriers, navigator services, bowel preparation details, and colonoscopy appointment specifics.

### Data collection and analysis

Table 1 describes the data sources used to capture PN program adaptations and lessons learned from navigators. Study team members, including PN trainers and qualitative researchers, collected data across multiple sources between August 2019 and April 2022. Data sources included trainer reflections after the delivery of the training program and qualitative debrief interviews.

**Table 1:** Patient navigation (PN) program qualitative data sources.

DATA SOURCE	DESCRIPTION	RESPONSIBLE PARTY	SAMPLE SIZE
**Patient Navigation Training Program Reflections**	Debrief notes after training sessions and technical assistance meetings to discuss overall impressions of the training, successes and challenges, levels of participant engagement, and need for modifications to content and format of the training session	Patient navigation program trainers (JHT, JSR)	9 navigators
**Patient Navigator Debrief Interviews**	Semi-structured, in-depth phone interviews with subset of navigators	Qualitative researcher (JLS)	3 navigators interviewed at two time points
**Review of Patient Navigator Interview Summaries**	Member checking review of interview findings to aid in thoroughness and trustworthiness of interpretations	Patient navigator (PG)	1 navigator (distinct from those interviewed above)

To track adaptations and lessons learned related to implementation support, the authors relied primarily on data collected by program trainers during debriefing sessions, and interviews conducted with patient navigators.

#### Training program reflections.

Adaptations to the PN training program were tracked by study staff, specifically the program trainers (JSR, JHT). The trainers debriefed after every training session to discuss overall impressions of the training, successes and challenges, levels of participant engagement, need for modifications to content and format of the training session. Trainers also considered the feedback collected during the debrief interviews with navigators (see below) for identification of possible ongoing modifications to the program.

#### Patient navigator debrief interviews.

The research team conducted two sets of interviews with three patient navigators to explore their experiences implementing the navigation program and identify ways the program could be improved. We interviewed the three primary navigators with the greatest duration of experience in the role (each with 9 months or more). The remaining navigators were not interviewed given their limited time in their role (6 months or less) or inability to participate in the interview process due to availability.

Study staff experienced and trained in qualitative methods (JLS) designed the navigator interview guide (see S1 File), conducted interviews, and analyzed transcripts content. Interviews explored barriers and facilitators the following processes: navigation training; contacting participants; partnering with GI practices; delivering the protocol and related topic areas; identifying resources for addressing colonoscopy barriers; and documenting navigation efforts. Interviews also explored navigator suggestions for improving the program and its implementation. Each of the three navigators was interviewed twice – once approximately 3 months into their role and again about 6 months later. This allowed us to identify early challenges that could be mitigated by program implementation adaptations, and to explore how barriers and facilitators to implementing the navigation program unfolded over time. Interviews were conducted by telephone, lasted 60 minutes, and were audio-recorded and transcribed. The transcripts were analyzed using a templated-based content analysis approach (see S2 File): JLS conducted multiple reviews of the interview transcripts to create summarized topic areas (e.g., barriers and facilitators to training; barriers and facilitators to tracking/documentation, etc.) [16–18]. Summaries were iteratively shared with study staff to inform adaptations to the program implementation. Additionally as a form of member checking our interpretations, one navigator who was not interviewed (PG) reviewed the summarized interview findings for thoroughness [17]. These summaries form the basis of interview findings presented in this manuscript.

## Results

Lessons learned from reviewing the PRECISE training program reflections and patient navigator debrief interviews were categorized into five main categories: 1) PN delivery, 2) implementation support and quality assurance, 3) staffing, 4) partnerships and resources, and 5) data tracking. Successes, challenges, and recommendations within each category are detailed below based on the integration of our PN training program reflections documented by study staff and navigator debrief interviews. Illustrative quotes from the navigator interviews highlighting successes and challenges within each category are provided below. Table 2 summarizes lessons learned within the five main categories, as well as recommendations for future implementation.

**Table 2:** Summary of lessons learned from implementation of a patient navigation program for follow-up colonoscopy.

LESSON/TOPIC	ADAPTATION/RECOMMENDATION	RATIONALE	IMPLEMENTED DURING PRECISE TRIAL	RECOMMENDATION FOR FUTURE PROGRAMS
**1. Patient Navigation Delivery**				
**Streamlining communication**	Reduce the number of navigation topic areas from 6 to 4 focusing on 1) first call and barrier assessment, 2) bowel preparation review, 3) colonoscopy check-in, and 4) colonoscopy results review	Reduce staffing demand and workloads by reducing the number of navigation calls and focusing on key touchpoints		✔
**Awareness of program**	Ensure introductory letter or communication about the program is sent to patients	Improve patient awareness and receptivity of initial and subsequent outreach attempts		✔
**Timeliness of outreach**	Improve timing and coordination of reporting to navigators of abnormal FIT result and initial call attempts	Ensure patients are reached early in the colonoscopy referral process to address barriers and prevent delayed outreach		✔
**2. Implementation Support and Quality Assurance**				
**Training Content**	Develop a COVID-19 training module for navigators with information on prioritization of delayed colonoscopies, COVID-19 policies for care, messaging to address patient fear and anxiety around medical care	Address the impact of the COVID-19 pandemic on colonoscopy availability and processes	✔	
	Develop training and scripting for navigators on how to communicate and partner with GI practices	Improve communication, responsiveness, and collaboration with GI offices in support of patient completion of colonoscopy		✔
	Augment training with more information on biology of CRC and medical terminology	Help navigators explain CRC and the colonoscopy procedure to patients and build rapport with GI practices		✔
**Training Format**	Convert in-person training format to virtual platform by developing a series of training modules including pre-recorded videos and live webinar sessions	Sustain navigation support for patients during the COVID-19 pandemic while satisfying social distancing requirements as well as restrictions on work-related travel	✔	
	Offer both in-person and virtual training formats	Flexibility in training modalities to meet navigator preferences and address structural barriers with in-person training		✔
**Technical Assistance**	Provide ongoing training support to sustain the program including daily/weekly technical assistance and booster trainings	Aid in managing caseload, tracking, and documentation challenges to promote fidelity of program implementation	✔	
	Develop a resource guide of local GI practices that includes front- and back-office contacts, hours, policies	Improve communication, responsiveness, and collaboration with GI offices in support of patient completion of colonoscopy		✔
**Quality Assurance**	Enhance training elements, including learning and practicing MI techniques and practicing hypothetical patient scenarios, with special focus on patients demonstrating hesitancy, concerns, and logistical or cultural barriers to undergoing colonoscopy	Ensure navigators develop the skills necessary to effectively engage with and support patients at various stages of readiness throughout the navigation process. The consistent weekly cadence of these meetings ensured that navigators received timely feedback and guidance.	✔	
**3. Staffing**				
**Number of Navigators**	Hire at least two navigators who can simultaneously implement the navigation protocol and work with patients	Improve reach by allowing outreach to occur on different days and hours, improve timeliness of outreach and documentation, and ensure continuity of care when one navigator is out of the office		✔
**Staff Transitions**	Improve transition plans when there is navigator staff turnover	Ensure that training and documentation files within the FQHC setting are easily accessible to facilitate seamless staff transitions and uninterrupted patient navigation		✔
**Increase Awareness of Program with FQHC Staff**	Improve awareness and understanding of navigation program among health system/clinic staff and providers	Improve communication and coordination regarding patient needs or questions that arise during navigation, such as delayed colonoscopy referral		✔
**4. Partnerships and Resources**				
**GI Practices**	Partner with GI practices to expedite referrals, improve scheduling processes, and better understand COVID-related policy changes, including the conversion of some pre-procedure consultations to a phone-based platform and COVID-19 testing requirements prior to the colonoscopy	Provide PN with resources to address system-level colonoscopy completion challenges (e.g., long wait times, referral delays)	✔	
	Continually build relationships and partnerships with GI practices and make this an ongoing process that requires contact updating and effort (**see Technical Assistance section above**)	Improve communication, responsiveness, and collaboration with GI offices in support of patient completion of colonoscopy		✔
**Transportation**	Apply and secure transportation grants (e.g., Lyft Community Grant) to offer ride credits for patients in need of transportation to their follow-up colonoscopy appointment	Provide PN with resources to address transportation barriers	✔	
**Funding for Uninsured or Underinsured Patients**	Partner with state and local health departments and other entities to provide funding for patients unable to pay for their colonoscopy	Provide navigator with resources to address cost barriers for uninsured and underinsured patients	✔	
**Identification of Ongoing Resources**	Continually research and identify resources available to patients, particularly regarding costs, companion, and transportation needs in rural areas	Identify new and relevant resources to overcome key patient barriers to colonoscopy		✔
**5. Data Tracking**				
**Database**	Modify REDCap® data tracking system to capture open-ended responses	Improve navigator’s ability to capture patient experiences and barriers that could not be properly tracked using discrete fields	✔	
**Tools**	Create one documentation and tracking system embedded within the FQHC’s chosen EMR	Reduce time and allow for improved integration and communication with the FQHC staff and other cancer screening prevention efforts		✔
	Embed key elements of script into documentation/tracking tool	Aid in thorough documentation process and serves as a reminder of key navigation topics to cover with patients		✔
	Include interactive calendar and reminder functions in documentation/tracking tool with automated functions to notify navigators when call topics or follow up outreaches are due	Help with caseload management and decrease likelihood of inadvertently overlooking a patient on the navigator calendar		✔
	Create a written manual of documentation instructions and provide repeat trainings on documentation	Facilitate consistency and thoroughness in implementation, tracking, and documentation of program		✔

### Patient navigation delivery

#### Successes.

Navigators felt that the introductory letter mailed to patients prior to formal navigation outreach increased patients’ openness to the initial outreach call. They noted that reaching out within a week of the participant receiving the letter was crucial. Once patients were reached for the initial call, follow-up outreach for subsequent calls tended to become easier as the navigators had established rapport, and patients were more willing to answer their phone and/or respond to voicemails. The flexibility of text messaging post-initial contact proved to be an effective and efficient means of communication for some patients, especially for discussing colonoscopy appointments and bowel preparation needs. Navigators found it very useful to check in with patients after their first pre-consultation appointment with GI to ensure patients understood the colonoscopy procedure and bowel preparation process. Navigators were able to use this call to clarify any misunderstandings and address any unanswered questions patients had. Navigators also reported that even when patients declined the full navigation program after the initial phone call, they often welcomed education on what the colonoscopy was and why it is important or needed. In general, the confidence navigators expressed in delivering the navigation topics, educating patients, and addressing patient barriers increased between their first and second interview, with navigators noting that it takes practice to become proficient in delivering the topic areas and identifying resources to mitigate challenges. Navigators reported that patients frequently expressed gratitude during outreach calls, expressing how helpful the navigation was for their colonoscopy completion, and that it impacted their confidence and willingness to complete any future colonoscopies.

#### Challenges.

Navigators noted that not all patients received the introductory letter, which was intended to enhance awareness and understanding of the program and outreach. Additionally, while they had some flexibility in working hours, most navigators had a typical workday of 8am-5 pm and felt that these hours hindered their ability to make outreach or follow-up calls during evenings and weekends when patients might be most reachable. The goal of making six call attempts for initial outreach often felt unrealistic and burdensome to the navigators, who also needed to prioritize time-sensitive outreach calls for patients already engaged in the process. Navigators also felt this number of attempts could be off-putting and intrusive to patients. Timing the outreach calls to the GI referral also proved challenging. Navigators sometimes postponed outreach calls to patients because of delays in placing the GI referral from the FQHC; at other times, the referral moved along so quickly that patients were already engaged in bowel preparation or had completed the colonoscopy by the time navigators made contact.

Navigators reported that they initially struggled to simultaneously utilize the calling scripts while also documenting in the tracking tool and felt the need to memorize call content. Coordinating with interpreters (for patients who did not speak English or Spanish) added another layer of complexity, especially when attempting to use motivational interviewing techniques, as the interpreter did not always represent the message the way the navigator intended.

Navigators reported that some patients declined navigation appeared to do so because they were familiar with the process and/or already had assistance from someone who was familiar. They reported that other patients declined navigation because of fear or lack of interest; cost or insurance concerns; competing health priorities; and pandemic-related concerns.

#### Recommendations.

Navigators suggested streamlining certain call topics, particularly the latter topic calls (topics 4–6), as patients were often occupied or resting on the day of their colonoscopy. They felt that combining topics 5 and 6 to focus on results would be acceptable to patients and reduce calling burden on the navigator. Navigators noted that some patients did not receive the introductory letter and felt that finding ways to improve receipt of outreach materials would help foster better receptivity to the navigation calls. They also recommended reducing the number of initial call attempts to alleviate patient annoyance and minimizing the number of outreach attempts for any call topic to help the navigator manage their workload while actively delivering the protocol to engaged patients. Finally, it was noted that FIT results did not always load in a timely way into the study tracking tool, thus delaying outreach – some patients had already completed a colonoscopy by the time they were contacted. Navigators felt that improving the timeliness of FIT results would allow navigators to reach more patients early in the colonoscopy referral process to provide education, address fears, help with bowel preparation and any needed resources.

### Illustrative quotes from patient navigators on patient navigation delivery

#### Success quotes.

*A lot of the times when patients are kind of like undecided about getting their colonoscopy done it’s because they’re just not very well informed. So, ask them if explaining the procedure or the reason why it’s needed like could help them. And then pretty much once I’ve done the explanation then they kind of make the decision on their own. And they realize why it’s important. I give leeway to ask them more questions because they’re unsure and then they’re pretty open to listening or talking about it.*

*And the text messaging, I just send them a text like, ‘hey, I know your colonoscopy is coming up. I want to make sure you have your bowel prep. Or let me know if you have any questions’. And then it will just be as simple reply, ‘oh, I got everything. Thanks.’ And it’s not like a big response that I’m expecting from them or anything. But it’s just letting them know that I’m not leaving them hanging or without support.*

#### Barrier quotes.

*It is hard when you have patients that won’t answer. I know I’m supposed to do like the sixteen calls for each topic and I feel like it can be a lot, like maybe one of these days they’re going to answer and they’re going to be so mad from all the call attempts, and because I still have to go through each topic.*

*One of the most difficult things is keeping in constant communication with the patients. Of course some patients have jobs they have to go to, other responsibilities. I’d say almost every patient there’s times where I can’t get in touch with them, and I have to leave a voicemail and get in touch with them a couple days later. And there’s sometimes problems with the communication where I can’t get in touch with them at all and then have to call them again and update them on all the information I have or just ask them what’s going on there since I haven’t heard much*

### Implementation support and quality assurance

#### Successes.

Navigators described the training they received as helpful and necessary to succeed and spoke highly of the training materials (e.g., navigation manual, call scripts, video training content, etc.). Those who received in-person training (prior to COVID-19) noted that they found this format particularly useful. Having access to updated videos and materials allowed navigators to refresh their training as needed. Specifically, navigators emphasized the usefulness of the scripting regarding bowel preparation and how to answer common patient questions, such as those about procedure reports vs. pathology reports. Additionally, navigators reported that training in motivational interviewing techniques was essential for “meeting patients where they are at,” aiding the navigator in honoring patients’ feelings and decisions without judgement, and fostering skills for managing resistant or fearful patients. Finally, navigators felt that having practice calls with staff and engaging in ongoing quality assurance activities bolstered their confidence and proficiency in delivering protocol content and working with challenging patients.

#### Challenges.

Navigators identified a few barriers in the training and quality assurance support. They noted that learning medical terminology and concepts related to colonoscopies and CRC could be difficult. Consequently, effectively conveying what a colonoscopy procedure entails (e.g., removal of polyps, or findings of adenomas, etc.) was sometimes challenging. Navigators also felt there was not enough training or practice on how to manage resistant patients and patients that were hard to reach. They also felt they lacked training on how to contact and build relationships and rapport with GI practices, particularly those less responsive to navigator outreach; this hindered effective communication and partnership for the benefit of patients. Given that there were more than 70 GI offices serving the referred patients, it became apparent that such a high volume of patients posed a challenge for one centralized navigator.

#### Recommendations.

Navigators recommended retaining all the existing training and quality assurance elements, including: practicing with hypothetical patients; learning motivational interviewing; in-depth in-person and video training on CRC, colonoscopies, and the intervention protocol; and quality assurance sessions. They also suggested adding modules and scripting on how to communicate and partner with GI practices, how to prepare for and manage difficult patient scenarios, and the biology of CRC and related medical concepts. Navigators suggested developing and frequently updating a resource guide of GI practices that includes direct back-office phone numbers and contacts for schedulers, nurses, and other key GI staff important for the navigator to partner with on behalf of the patient. They suggested that navigation services should ideally be regional (rather than centralized at a single clinic location) so navigators can build relationships with a smaller number of local GI clinics and learn their processes and resources.

### Illustrative Quotes from Patient Navigators on Implementation Support and Quality Assurance

#### Success quotes.

*I thought the scripting was very helpful. Like when it came to topic three and you have a whole script about what you’re supposed to talk about with topic three. I thought the question, the Q &A that was provided to us, like the answers to like questions that patients might have was really helpful … And also something I found really helpful was just the constant check-ins.*

*So, after the quality assurance check in, staff would tell me little things, like it would be nice if you asked such and such or that probably wasn’t the best time because I could hear kids crying in the background or something. … before the QA policy of just practicing, we did like for ten patients. And [Name] would be like a difficult patient, or sometimes she would be like just someone that was fine with it, going through with it. So it was nice to get that feel for what I might expect.*

#### Barrier quotes.

*They did mention there may be some conversations here and there that may be tough to go through like if someone is starting off with cancer. I believe having mock calls with staff regarding those topics would be great to have some experience, as my first conversation I was caught off guard. Of course I tried my best to listen to the patient and go about it as appropriately as possible, but I do believe having a bit more training on those type of conversations in the beginning would be helpful.*

*Some [GI offices] are easy to work with and some are not. I think when it comes down to medical records, I have to be careful with my words and terms, because I am not a provider, but I am a care coordinator. More training on how to communicate effectively with GI would have helped.*

### Staffing

#### Successes.

Navigators described that being bilingual in English and Spanish and having a customer service background were both important skillsets to bring to the navigation role. They reported that working in the clinic alongside other clinical staff facilitated communication and care coordination for navigated patients. Navigators found it particularly helpful to participate in weekly meetings or team huddles with the FQHC’s cancer prevention team. Given their large caseloads of patients, the navigators found it helpful to receive support from other staff or interns at the FQHC to deliver the program introduction and initial 18 outreach attempts. This support allowed navigators to better manage their engaged patients through the colonoscopy process, focusing on support, education, and barrier mitigation. Finally, navigators appreciated being able to meet with the research staff on occasion to learn about the overall status of the program, address any challenges, and identify what further efforts were needed to meet recruitment and engagement goals.

#### Challenges.

Navigators noted that having only one full-time navigator was sometimes insufficient to manage all the patients being actively navigated while also engaging in initial outreach to newly eligible patients. This, coupled with staffing turnover in the navigator role and within the FQHC more broadly, reduced the likelihood of reaching a patient in a timely manner and meant that some patients were not contacted on time for their outreach steps. Navigators noted that navigator staffing transitions were often challenging because training and documentation files within the FQHC setting were not easily available, hindering a seamless transition for patients being navigated.

FQHC staff responsible for sending the introductory letter did not always send it in a timely fashion, if at all. Staff were also inconsistent in entering abnormal FIT results into the intervention tracking system, leading to delays in outreach. Navigators observed that staff at both the FQHC and GI offices were often unaware of the intervention, which sometimes hindered communication and coordination with providers and specialists. Low program awareness within the FQHC also hindered warm hand-offs or responses to patients who called the health center with questions about the program. Finally, during the COVID-19 care suspensions, there was low staffing at both FQHC and GI offices, which often delayed both scheduling and obtaining results of colonoscopies.

#### Recommendations.

Navigators strongly recommended having at least two navigators simultaneously implementing the navigation protocol and working with patients. They felt this approach would maintain a manageable caseload of 30–50 patients per navigator, reducing delays in outreach or follow-up efforts. Two navigators would improve reach by allowing outreach to occur on different days and hours, improve documentation, and ensure continuity of care when one navigator is out of the office. Multiple navigators would also aid in the efforts to reach the many GI clinics and/or facilitate maintaining an up-to-date GI resource list. Navigators also suggested improving awareness of the navigation program among FQHC staff and providers, as well as GI staff and specialists, to improve coordination. Finally, navigators highlighted the need for better transition plans when a navigator leaves and a new one assumes the role.

### Illustrative quotes from patient navigators on staffing

#### Success quotes.

*Coming into this position, I worked in insurance beforehand, so bill keeping that did overlap with my previous position. And this current position now has a lot of clinical customer service, of course this is not technically customer service, but a lot of talking with people, understanding where they’re coming from and their issues. In this case I’d say my background and the training was definitely great in terms of getting me to feel confident and prepared to have these conversations with patients.*

*It’s nice to have like a few people [at FQHC] working on the same patients. Like, care coordinators here [at FQHC] we have three or four for all the cancers. And just knowing that there’s more than one person looking into their chart because I’ve found that some of the patients I’ve worked with have been getting good follow up with the medical assistants and the provider on getting this process done, even just getting enrolled with the community financial service for their colonoscopy. So just closing those little care gaps.*

#### Barrier quotes.

*It would be nice to have someone, like at least for the beginning and sit in the end of the week when it gets a little bit more saturated, when I’m getting a new batch of patients and having to review them. And at the same time at the end having to close out other patients. That’s usually where I struggle, at the beginning and at the end of the week. For example, if I had someone that could help do the check-in call before the colonoscopy, just those more time sensitive cases that I need to make sure I reach out to – it would be nice to have more staff for those.*

*There were times where I would run into a patient’s chart, and I am trying to do all those first navigation steps. And I don’t see a referral in there, even if they had a FIT test, so I have to make a telephone encounter in Epic and I have to talk with the provider and be like, ‘hey this is who I am, this is what I do. I am working with this patient, and I see that you ordered a FIT test for this patient on this certain date, but I don’t see the referral can you please have someone on your care team follow up on the patient and assign a referral so that we can get started with the process’. And sometimes because of staff and coordination that was taking a month or a little bit more for MAs or providers to answer those telephone encounters.*

### Partnerships and resources

#### Successes.

Navigators emphasized the importance of building and continuously nurturing rapport with GI practices, including explaining their navigation role, and learning the GI office’s workflows, policies, and resources (e.g., for transportation or costs). Navigators noted that this rapport increased responsiveness and support from GI practices. During the COVID-19 pandemic, navigators recognized how reassuring it was to patients for navigators to be able to explain and facilitate the COVID-19 related requirements of GI practices, such as testing or vaccine requirements, prior to the colonoscopy.

Being able to offer and help coordinate rides from the Lyft grant was also perceived as helpful, particularly when patients experienced last-minute changes in their planned transportation. In some cases, navigators served as a companion or ride option for patients when other resources were unavailable. The obtainment of new charity care resources (e.g., CDC grant) allowed navigators to offer more flexible and efficient financial assistance options for patients needing to complete their colonoscopy.

#### Challenges.

Navigators noted that some GI clinics were very difficult to coordinate with. Some GI practices would not respond to the navigators’ calls or faxed requests on behalf of patients. Some GI practices were unwilling to share information about navigated patients’ pre-consultation or colonoscopy appointments or would not allow navigators to schedule appointments on behalf of navigated patients. Navigators reported that GI offices were sometimes unclear in explaining to patients the difference between a pre-consultation appointment and the actual colonoscopy, and some practices did not provide clear bowel preparation instructions. Navigators noted that smaller GI facilities tended to be less well staffed and resourced, lacking interpreters and companion/transportation support. Complicating this, some GI facilities would not book a patient’s colonoscopy without a confirmed ride or companion, while patients desired to book before verifying their companion/transportation support, thus often creating scheduling delays. The Lyft option for navigated patients did not always work well as the service had limited or no drivers in rural areas where some navigated patients lived, or patients were uncomfortable with this transportation option. Some GI offices did not have resources for those who couldn’t pay, or had long, burdensome applications for financial assistance.

During the COVID-19 timeframe, GI practices were often closed or had limited hours and staffing, which exacerbated the challenges of reaching GI staff discussed above. Additionally, GI offices varied in their pandemic-related policies, making it difficult for navigators to relay accurate information to patients about these policies.

#### Recommendations.

Navigators stressed the importance of finding ways to build rapport and open communication channels with GI practices. They felt this should be an ongoing process, involving consistent contact and education of GI practice staff– including front office staff, nurses, medical assistants, schedulers, and GI specialists – on the PN program. Finding additional financial resources for patients with simplified application processes was also noted by navigators. While partnering with a ride service like Lyft was perceived as useful to navigators, this was only helpful when the geographic range and availability of drivers covered the areas servicing navigated patients. Overall, navigators desired more resources to support patients in securing companions, transportation, and covering costs, particularly for patients living in rural areas.

### Illustrative quotes from patient navigators on partnerships and resources

#### Success quotes.

*Once I am touch with the GI office and speaking with them it’s been pretty good so far. They’re all professional and understanding, and I’d say with the GI clinics that we’ve partnered so far, it’s actually been a great experience.... They’re on top of things 110%, so if I refer them a patient to send all the information they need and they’re pretty great about keeping me updated as well. With the GI clinics, the ones that we’re not partnered with, I constantly have to check in on them since of course they have so much they have to do so they can’t reach back to me efficiently.*

*There’s a pot of money set aside for patients who are under-insured or not insured at all to get their colonoscopies covered and it pretty much covers everything for the patient. I just had my first two patients use that grant within the past two weeks, which is great. And one of those patients they were really reluctant at first because they were first given the financial aid application and they were confused by it, they were like, ‘I was told it was going to be covered, why do I need financial aid?’ So thankfully we do have that grant, it has helped a lot of those patients who don’t have insurance and need to cover the cost.*

#### Barrier quotes.

*Even if I tried, the [GI office] won’t schedule with me – some won’t let me schedule the appointment for the patient … I’ve also had patients that are confused and they’re thinking that they’re going for their colonoscopy. And then they’re worried because they don’t have instructions, which most likely it’s that it’s been a pre-consult but it’s not very clear what they scheduled from the GI office. So, I’ve learned to double check with patients. As soon as they give me a date for their appointment, or even before. I ask, ‘Okay, when they schedule you make sure that you check if this is a consultation or if it’s the actual procedure.’*

*The Lyft [transportation option] we do it through a website through the grant that we got. I’m just going to check Lyft to see if the ride went through. And I checked our Lyft website, and it said that no drivers were available at that time. And I don’t know if it’s because it’s in a more rural area that there’s just not as many Lyft drivers accessible. So maybe we need to be more careful where and when we’re scheduling and if there’s even like Lyft drivers in that area.*

### Data tracking

#### Successes.

Navigators appreciated that the REDCap tracking system used for documenting call attempts and navigation topics also helped served as a helpful memory prompt for important content to cover in the calls. Additionally, navigators appreciated the tracking calendar of needed calls for each participant and the ability to review this calendar with research staff. This aided navigators in organizing their calling time and efforts and keeping their outreach calendars up to date so as not to inadvertently overlook any patients in need of initial outreach or a specific call topic, such as a bowel preparation check in call.

#### Challenges.

Navigators found it confusing and time-consuming that actions had to be tracked and documented in the REDCap tracking database for the study, and within the FQHC’s electronic medical record (EMR). Additionally, they reported that some navigators did not provide enough detail in their documentation for others to easily take over if needed. Navigators also noted that some potentially useful reminders and prompts within the REDCap tracking were “buried” in the tracking system, and hence sometimes missed or forgotten.

Just prior to the onset of the COVID-19 pandemic, the FQHC transitioned to a new EMR platform, but efforts to train staff on the system became diverted to COVID-19 tracking needs during the pandemic. At times, lack of expertise in the new EMR prevention, screening, and referral workflows made it harder for the navigator to obtain accurate patient data relevant to their colonoscopy and medical history.

#### Recommendations.

Navigators strongly recommended creating one documentation and tracking system, and having it embedded within the FQHC’s chosen EMR. This would reduce time and allow for improved integration with other cancer prevention efforts and communication with FQHC staff. Navigators also suggested that key portions of the script representing different call topics be embedded within the documentation and tracking tool to facilitate both patient education and documentation. Additionally, navigators recommended the tracking tool have an interactive calendar function and the ability to automatically notify a navigator when they have a new patient on their caseload or an active patient who needs a topical outreach call. To improve timely outreach when navigators’ caseloads are larger, the navigators suggested the tracking system send reminders to navigators regarding active patients that have not been contacted recently to avoid having patients “lost” to follow-up. Finally, navigators recommended having clear, repeated training and instructions on how to use the tracking system to aid in maintaining navigation and documentation consistency across navigators.

### Illustrative quotes from patient navigators on data tracking

#### Success quotes.

*Regarding workload with the patients and protocols, it’s been pretty manageable. I like to keep my calendar and schedule very organized to deal with the amount of the patients that can be a lot at times. But it’s nothing too worrisome for me, I feel like I can handle it as long as I am organized and keep myself in line as I usually do. So we use REDCap to keep track of all that data. I do have worries sometimes where one patient may fall through the cracks and may not be reached out to in a while. So that’s when I started checking my call tracker and literally going through every single patient I have, every Monday to make sure I reached out to them this day and this time, and I have to reach out to them a couple days or so whenever their next scheduled call is. That way I am aware that all my patients are being reached out to and helped and no one is falling through the cracks.*

#### Barrier quotes.

*I feel like documentation is a lot … just because everything with the EMR and that there’s so many places that you can document things. So if you can keep everything noted in one spot and make sure it reflects the pertinent patient information needed, just consolidate those things so that you’re not doing the work over or having to like review extra charts just because you’re not sure if things were done or where you left off across tracking tools.*

*So it was kind of like two things hitting us at once, a new medical record transition to EPIC and COVID. I feel like in terms of EPIC it was harder because we were trying to figure out how will the centralized cancer team order FITs? You know, there’s not like a lot of data that we’re able to gather. And I think also just our data analysts being like overwhelmed with work due to the transition and COVID. And that kind of also affected our weekly reports for navigation outreach. Because you know, we needed certain reports to be fixed if they weren’t running properly.*

## Discussion

Through a review of PN training program reflections and patient navigator debrief interviews, we identified a number of key lessons from a PN program for follow-up colonoscopy delivered as part of a pragmatic clinical trial at a large FQHC during the COVID-19 pandemic. Key lessons learned include:

1) improving **patient navigation delivery** through streamlined navigation topics, enhanced introductory program material, and timely patient outreach,
2) retaining existing **implementation support and quality assurance** elements, but adding new modules on communication and patient management,
3) **staffing** the program with at least two navigators to manage caseload and emphasizing the need for smooth transition plans when navigators change roles,
4) developing and strengthening **partnerships** with GI practices to facilitate colonoscopy appointment coordination and identifying more **resources** to support patient barriers, and
5) creating a unified documentation and **data tracking** system within the FQHC’s EMR to ensure consistency and efficiency in patient navigation and documentation.

These findings inform the development and implementation of future PN programs for colonoscopy and other preventive care measures.

Overall, our study adds to the growing body of literature on barriers, facilitators, and guidance for PN programs. Consistent with Chan et al.’s systematic review [19], we identified challenges with EMR systems and the importance of well-developed training processes and proper navigator supervision as key organizational-level facilitators to successful program implementation [19]. Our findings also align with DeGroff et al.’s [20] emphasis on understanding the target population, as our bilingual navigators noted the value of cultural competence in care coordination.

Building strong partnerships with GI practices also emerged as a crucial factor in facilitating communication between primary and specialty care, addressing scheduling barriers, and identifying financial resources for underinsured or uninsured patients. This aligns with Kokorelias and colleagues’ scoping review [21], which highlighted the importance of appropriate caseload management for program sustainability and quality of care. Caseload was a key limitation identified in our study: navigators shared challenges with the number of patients they were assigned and noted that a high number of call attempts can be difficult to accomplish and intrusive to patients. In a separate analysis completed by our team, we found that 93% of navigated PRECISE patients were reached within six calls, and tripling the number of call attempts (up to 18) resulted in low yield of reached patients. Furthermore, we found navigated patients reached within six attempts had higher colonoscopy completion rates within 12 months of an abnormal FIT than patients reached between 7–18 calls (61.2% vs 30.8%, respectively) [22]. These findings indicate a point of diminishing returns at six calls, corroborating navigators’ impressions.

### Strengths and limitations

One of the strengths of our program was the provision of ongoing training and support throughout the program, including through the COVID-19 pandemic. Our research team learned to be flexible and resilient during this time period, making training adaptations based on a rapidly changing context. Although the in-person training was a rich and engaging experience, the adapted virtual platform allowed us to offer education to additional FQHC staff and address staff turnover. Furthermore, a virtual training allowed for a shorter time commitment, elimination of travel time for training, and satisfaction of COVID-related distancing requirements. The hybrid model of pre-recorded videos and interactive virtual classes catered to different learning styles and provided complementary approaches to learning. The adapted virtual training could also be used for booster training purposes for existing and new staff.

However, there were some limitations to the PRECISE study. First, it took place during the COVID-19 pandemic, which likely introduced unique challenges and may have impacted implementation and results in ways that would not apply in non-pandemic times. Second, staff turnover created inconsistent navigation at times. Third, the relatively short study duration of two years may not capture longer-term impacts or sustainability of the navigation program. These limitations should be considered when interpreting the learnings and considering the applicability of such findings in other patient populations.

### Future research

Future research should focus on developing an integrated and efficient data tracking tool essential for optimizing CRC screening follow-up navigation. This tool must seamlessly integrate with the EMR, enabling real-time monitoring of key program outcomes and streamlining patient management through automated reminders, follow-ups, and scheduling. Additionally, research should explore strategies to minimize navigation touchpoints while maintaining effectiveness, such as reducing program contacts while utilizing advanced technologies like video messaging. By combining an enhanced tracking tool or database with optimized navigation strategies, future studies can strengthen CRC screening follow-up programs, improving both efficiency and patient outcomes across diverse healthcare settings.

## Conclusion

In conclusion, the review of patient navigator training program reflections and debrief interviews from a pragmatic clinical trial at a large FQHC during the COVID-19 pandemic has yielded invaluable insights into enhancing patient navigation for follow-up colonoscopies. Key lessons include improving patient navigation through enhanced introductory materials, streamlined topics, and timely outreach; retaining and expanding existing training and quality assurance elements; ensuring adequate staffing with at least two navigators and smooth transition plans; developing strong partnerships with GI practices to address patient barriers; and implementing a unified documentation and data tracking system within the FQHC’s EMR. These findings are crucial for informing the development and implementation of future patient navigation programs, not only for colonoscopy but also for other preventive care measures, ultimately aiming to improve patient outcomes and healthcare delivery efficiency.

## Supporting information

S1 File Navigator interview guide. (DOCX)

S2 File Interview summary extraction. (DOCX)
