# Supplementary material for: Implementing a successful patient navigation program for follow-up colonoscopy: Lessons from the PRECISE study
Source: PLoS One. 2026 Mar 18;21(3):e0343659. doi: 10.1371/journal.pone.0343659 (PMC12998853; doi:10.1371/journal.pone.0343659)
Supplement: S1 File — (DOCX) [file pone.0343659.s001.docx]

**SUPPORTING INFORMATION FILE 1**

**PRECISE STUDY: Patient Navigator Interview Guide – conducted via phone**

1. Please describe your role at Sea Mar.
2. How would you describe your role as a patient navigator (PN)?
   1. How does your role as a PN support the PRECISE study?
3. Overall, how has the PRECISE study been going? [*ask open ended*]
4. What have been some challenges with the PN intervention work from your perspective? [*ask open ended then probe on below]*
   1. Reaching the patient
   2. Documenting/ reporting/ tracking
   3. Time management
   4. PN protocol deliverables
   5. Work schedule
   6. Training back-up / or not enough training
   7. Identifying patient resources
   8. Encouraging patients to complete their colonoscopy
   9. Navigating the follow up colonoscopy process with GI specialists and communicating with clinics
   10. Answering questions about insurance/ signing patients up for insurance
   11. Integrating with other work/roles at clinic
   12. Other/ anything else
5. Based on your experience delivering the intervention so far, what are some areas for improvement/changes? Or, what could be done differently to improve the program? [*open, then probe below*]
   1. Are there calls or topic areas that could be combined or streamlined in some way?
   2. Are there other supports or resources you need to help you in this role and assist patients?
      1. How well did the training help prepare you for your role? Helpful or not? Any needed changes or improvements to content, frequency, etc.?
   3. Are there other supports or resources you feel patients require to help them complete the colonoscopy – is the program missing any topic area/support from your point of view?
6. What has allowed you to be successful in your role as the PN? [*asked open ended then probe on below*]
   1. Check-ins with manager
   2. Quality assurance (QA) process
   3. Protocol
   4. Training or refreshers [*if not covered above: prompt on if received enough training/need more/ need a different focus or if it was just right, frequency of trainings, how to improve training, etc.*]
   5. Communication style of team
   6. Meetings
   7. Support/interaction from other staff at clinic
   8. Other/ anything else
7. How do patients typically react to the PN outreach? Please describe [*probe for both positive and less than positive examples*]
8. From your experience, what call/topics have patients found most helpful? Why do you think that is?
   1. What call/topics have patients found least helpful or confusing? Why do you think that is?
9. From your experience, when do you have the best chance of reaching patients?
   1. How do you structure your work schedule to help with patient reach?
   2. What sort of calling strategies have you implemented to help with patient reach?
   3. Have you noticed a patient preference for live call, text, etc. for the colonoscopy process?
10. What have you heard from patients regarding barriers to colonoscopy?
    1. How have you been able to resolve barriers?
    2. What gets in the way of resolving colonoscopy barriers for patients? If you were not able to help resolve the barrier, what did you do? Please describe.
11. Were your interactions with patients primarily over the phone or did you ever meet with a patient in person?
12. Overall, do you feel the PN program is helpful to patients – if so how? If not, why not?
13. Overall, do you feel the PN program is helpful to Sea Mar – if so how? If not, why not?
14. Anything else you would like to share or let us know about it from your experience so far in the PN role?
