# Supplementary material for: Implementing a successful patient navigation program for follow-up colonoscopy: Lessons from the PRECISE study
Source: PLoS One. 2026 Mar 18;21(3):e0343659. doi: 10.1371/journal.pone.0343659 (PMC12998853; doi:10.1371/journal.pone.0343659)
Supplement: S2 File — (DOCX) [file pone.0343659.s002.docx]

**SUPPORTING INFORMATION FILE 2**

**PN Summary extraction template:** *Based on interview guide and iterative review of transcripts, topical areas were defined along with summary points from the interviews. Illustrative quotes that support these findings were extracted from interview transcripts and listed at the end of the document.*

**Training/preparation**

*Facilitators*:

Pilot and practice; training was thorough and sufficient; study staff very quick to respond if or when have any questions

QA with staff very helpful esp for first set of patients navigated/ plus doing practice calls with study staff as part of QA also helpful

Actually visiting GI offices helpful to learn their process, what to expect and build rapport with them

Training was good/helpful – including QA and MI, creating a PN manual that is electronic to track updates and changes plus excellent scripting for explaining bowel prep or answering other questions from patients

PN prior experience in customer service with insurance/ ease of talking with ppts was helpful, even though no medical background exp prior

Being bi-lingual is essential

The more you do the navigation, easier it is to delivery topics, manage # of pts, etc / become more proficient and organized over time

*Barriers or pain points*:

Training was sort of long, but also necessary

Due too COVID inability to have face to face, in person didactic training which is how PN best learns / harder to learn via just TEAMs and videos

PN has no medical background or training so finds learning medical terms, esp pertaining to colonoscopy and results, difficult

Not totally prepared for hard conversations such as if ppt had cancer when PN called/ felt needed more practice QA mock call practice with Jen R on “difficult scenarios”

*Suggestions*:

Needed more medical terminology training/understanding esp if lack medical training / More training over time on “biology” of colon cancer so can better answer patient questions on this topic and sound educated and informed versus saying “all get back to you, don’t know that”

Felt needed more practice QA mock call practice with study staff on “difficult scenarios”

**Reaching patient**

*Facilitators*:

Once reach for first call and can establish relationship and ability to text, then it becomes easier to reach ppt, plus familiar with ph # after first call/ plus just reminding ppts how important it is to stay on top of health even during COVID

Texting is often a ppt preference or text prompts response or call back from ppt/ text option continues to be helpful esp for calls 4-6 and engaged ppts often text or call back from message

Try to get first call within a week, calling at multiple times, after hours, and once on Saturdays if needed; monitor times when have tried to reach ppt successfully or not and use that as a guide for future calls for PN topics after first outreach; does notice tends to reach more ppts in the evening for the first call/introduction/ also really tries to keep calendar up to date and reviewed each week to stay on top of which ppts need which calls

Having intern make all the initial outreach calls (multiple attempts, VM’s) free’s up PN’s time to focus on ppts in navigation flow rather than trying to carve out time for both new calls and the # of required attempts and reaching ppts for designated nav call topics / once reached by intern then transfer call to PN for first intro

Letter helps – for those patients that recall and read it/ helps with reach and attempt- letter helps orient and educate ppts

*Barriers or pain points*:

Hard to reach patients, esp for first call when don’t recognize phone # - leave lots of messages and may miss return call from ppt or end up playing a lot of phone tag/ plus COVID fears, don’t want to leave house or is caregiver so fear to brining COVID home/ After COVID reaching ppts still hard, some just don’t answer or want to verify DOB, etc. (suspicious) nor respond to VM also find topic 5 hard to reach because it is day of colo

PN not always free or available when ppt prefers to talk or can speak

Can’t reach, hard to engage ppt and can become annoyed with so many attempts / hard to try 16 times, not reach ppt and then also fit in all the 6 topics under certain timeframe/ the 6 call attempts for first outreach in one week can feel intrusive towards ppts who are receiving texts or clear VM’s and choosing to not call back/ continues to be a time burden on PN

Some ppts reached have already completed colo (provider referral and GI office/opening happens quickly) or are in process of doing prep etc. Sometimes there is delay in outreach due to clinic not processing referral very fast/ including ongoing problems with FQHC MA’s not faxing referral correctly, hence creating delays/ some providers slow to fax referral and he has to send messages to them or their MA to fax/approve referral to GI, so delays the outreach

Noted some patients becoming more open to outreach/calls once COVID vaccines were available / explain all the protocols GI practices are doing to keep it safe or if ppt has had vaccine more open, but still seeing resistance in early 2021/ Eventually COVID less of a reason to decline colo and some GI offices less strict with requiring vaccine or testing or more flexible on type of covid text, not really an issue for ppts now

*Suggestions*:

Wonders if 6 attempts on one week is too much when getting letter, and VM’s/ maybe consider lessening this #/ when managing many active and engaged ppts, want to focus on them more than many attempts to non-interested ppts

Improve timing/timeline between learning about abnormal FIT and reaching ppt quickly so can offer navigation before they start or complete the colo on their own

Not all ppts want texting or can managing texting, so have to rely on calls and messages and may still not respond

**Delivering PN Topics**

*Facilitators*:

Pilot opportunity to practice with patients and topics; scripting and motivational interviewing training; having redcap set up in way to follow topics

Explaining why colo is needed or how procedure actually works often lessens fear and opens ppts up/ patients find this education piece very helpful

Patients find check ins and having PN find out info/communicate back to ppt very helpful, including scheduling, preconsult and check-ins on prep/ making sure to check in with ppt after preconsult very helpful to confirm their understanding or answer any questions they didn’t ask during preconsult/ plus explaining to patient any COVID testing requirements from GI and offering LYFT option

Ppt seem more confident and willing to due a future colonoscopy for their next surveillance/ also more open to talk about colonoscopies and “that part of the body” and following thru on next surveillance

Texting allows more flexibility – can let ppt know they can call or text during prep or other times if question comes up/ easier to reach PN this way then a GI clinic – allows for quick check in or getting answers to questions efficiently

Increased confidence/comfort level with topics/motivation as help more ppts/becomes easier

Honoring patient choice/meeting them where they are at without judgement

*Barriers or pain points*:

Initially hard to recall all the questions and content per protocol for each topic, try to memorize it but often had to navigate between script and redcap at first which was a little tricky

Reasons to decline service often because familiar due to prior colonoscopy and/or familiar and don’t want so many calls/outreaches by PN / some ppts decline because they are familiar or have helped someone else with colo or they ignore outreach attempts and never respond

# of call attempts and topic areas can feel like too much for ppts or like PN is bugging ppt/ same see above in reach

Covid fears for self or caretaker for family member/ don’t want to travel or leave home due to Covid / harder to find companion and cost concerns coming up more

**Assessing/helping with Patient Colo Challenges** (scheduling, escort/rides, fears, prep)

*Facilitators*:

PN sometimes able to serve as escort/transportation for ppt

Easy access via phone or text to PN for ppt to ask questions esp when in midst of bowel prep/ plus VM and repeated outreach to make sure they understand diet, timing of when take prep, notices some GI offices do not go over this well in detail, don’t offer instructions in other languages, non-English speaking ppts tend to need the most support re prep

Navigating information on lack of referral between clinic and GI and informing pt/facilitating it/ will ping PCP or MA if need be, notices delays in referrals much improved now out of COVID, only gets “bad” again during surges

Offering Lyft/rides helpful / developing relationship with GI’s so can learn their resources for escort and transportation/ but hard to find escorts still or sometimes get GI to respond to PN calls – uses Own LYFT account to order for pts as easier to time to end of colo/ but transportation and escort tend to be a big barrier still for ppts including being booked by GI clinic too far away from home, PN often google maps this and finds a closer GI facility for ppt so escort/transportation and travel time (over an hour for some) can be less burdensome

Encouraging/explaining to ppt to schedule colo and not delay scheduling it even if escort or ride is a bit unclear, as this is something PN can help them problem solve including with Lyft grant/ plus re COVID explain how safe clinics are with social distancing, disinfecting, masking and testing to try to help ppt overcome COVID fears for colo/ encourage ppts to be recontacted in 3 mo’s

New charity care option that is quicker and more flexible than traditional charity care applications / Charity care by GI, project access or new grant $ all options for under insured or noninsured ppts, finds the new grant is working well esp since project access often denies due to income or financial aid applications are overwhelming for ppts to do or figure out

*Barriers or pain points*:

Transportation, escort big challenge as is insurance/payment and use of BCHHP or project access / more uninsured patients with challenges for paying even if obtain a discount/ application process for financial assistance burdensome and time consuming for ppts to complete/ continues to be issue during COVID esp since PN can’t serve as companion or harder to find someone to go with ppt due to COVID/ plus LYFT not always reliable during COVID with more limited service option in rural type of areas

Misunderstanding preconsult versus colo or when/how to start bowel prep

Trying to deliver topics and use MI while using an interpreter is difficult – the 3 way conversation to try to motivate and education is hard

Long waits to schedule – 2 to 3 months; GI offices “not partnered with” re Coronado outreach tend to be harder to get to respond to PN for follow up info like when it’s booked or what is required re covid/ impacted by lack of staffing at GI offices due to covid outcomes

Have to schedule Lyft in advance so hard to time appropriately to end of colo and when ppt is ready to be picked up/ hard to coordinate with GI on this

*Suggestions*:

For LYFT need better understanding where drivers are less available due to COVID or rural area – better list of the real resource available

**Documentation and tracking/ schedule coordination**

*Facilitators*:

Documenting in redcap pretty easy, not a challenge to navigate it, makes sense to PN; the Redcap lay out goes with the flow of the protocol so serves as a ‘memory guide’ as well

Training intern/others how to document well in redcap so clear “where you left off” with ppt

Going over calendar and who is due for what every Monday to set week and what needs to be done, very important to keep calendar up to date for delivering PN to many ppts

*Barriers or pain points*:

Once start to get a lot of patients on que/schedule it becomes harder to track all the different type of calls for whom and to get all the calls in according to protocol; have to document in 3 different places and it is time consuming (redcap, EPIC and spreadsheet)

Prompt/reminder to ask if can text during call ½ is buried far into RedCAP so sometimes forget to ask this permission

During PN transition hard for CHR to see/know all the data in FQHC own tracking system so harder to get new PN’s up to speed on status of patients

Not always getting FIT results back quickly due to staffing turn over at FQHC lab, so delays in getting positive FIT results/ made worse during COVID

Transition to EPIC at beginning of COVID so hard to train on it and hard to access needed data as attention was on COVID and tracking that – made access to data and needed info for program harder

*Suggestions*:

Need 2^nd^ person as backup, esp at beginning and end of week (M ad F) to assist with closing out patients and or documentation or calling – hard to reach everyone when, esp for more time sensitive topics when, new patients loaded every week/ need more than one PN so can reach the # of patients out to be about 30-50 per navigator and give navs more time to reach ppts and be attentive to their needs

Need RedCap to notify PN when you have people to reach out to or if haven’t reached out to someone in a while for a double check of not losing people when have many on call list at different levels of outreach

**Coordination/Communication with GI practices**

*Facilitators*:

Helping patient schedule appt, do a 3-way call with ppt, PN and GI office; or offering ppt info re GI phone numbers or confirmation referral has gone thru to book it/ same

PN learned to always double check with ppt whether scheduled appt is preconsult or actual colo, helps ppt understand the difference between these two and coordination of them

Learning GI’s resources they offer for transportation/escort options/ some GI offices more helpful than others, larger systems seem more resourced than smaller GI offices

Serve as translator or set up interpretation services for patient with GI office/ assure this

Calling to find closer GI to ppts home, very helpful to ppt

*Barriers or pain points*:

Some patients want to call GI and schedule themselves and have PN call back in a few days after that, but then PN might struggle reaching the patients

GI won’t book colo often if ppt doesn’t have an escort/transportation option lined up at time of scheduling and this hard to patients, may take time to figure this out

GI office won’t allow PN to schedule appt on behalf of ppt, need to have ppt on the line and some ppts don’t want to, just want to book themselves

Clinics vary in approach/policies and how well they explain prep: one clinic won’t communicate or release info about ppt to PN over phone so PN has to communicate via faxing and they tend to not respond / prep instructions better at some GI’s than others

GI offices not very good at informing ppt of difference between pre-consult and actual colonoscopy and ppts often show up to pre-consult thinking it is colo

GI offices closed or with limited staffing during early COVID / Trying to determine each GI’s requirements re COVID testing as there was no standardization, some 24 hours, some 72 / then communicating this to ppt and helping to facilitate

Some GI offices harder to work with than others, don’t respond after multiple attempts to have referral re-checked or results re-faxed, feels some don’t respond to PN because as lack understanding in how to talk about or use medical terms/ easier with “partnered GI clinics” that non partnered clinics/ can be hard to get GI clinics to respond call back, easier with partnered clinics

**Coordination with FQHC**

*Facilitators*:

Sit with quality team for cancer prevention, participate in weekly information sharing huddles/ sit close to others on team along with referral coordinators

Mtgs with CHR staff to review status/numbers/goals always very helpful

*Barriers or pain points*:

Lack of referral to GI / delayed referral / PN has to re-do it or reach out to MA to have it redone – tend to be missing the abnormal FIT result to go with referral for GI to process

PN transitions and needing to train up for role

FQHC not always getting FITs out at mailing or point of care during COVID/ lab slow in processing results during COVID/ not much preventive care happening in early to mid-covid

*Suggestions*:

Need more awareness and education of clinics/providers that the precise program is going on / esp would help with any future support of keeping the program going for providers to be aware of service and understand how it is helping ppts get their colo’s

**Patient Reactions to PN/outreach**

*Facilitators*:

When reach patients often open to navigation, just need to build that early rapport and relationship in first call; a lot of patients appreciate the program and support

Family member or friend drive/escort

Prep isn’t as bad as patients think it is once PN explains it (myth can’t eat at all or super strict) plus some GI practices have really good food explanations she can share/ focus on WHAT you can eat rather than just what you can’t eat/ ability to text or call PN in the moment of prep very helpful

Patient will call or text PN when doing prep to clarify any questions or ask if made mistake

Ppt’s even when resistant, or declining may still be open to education and learning reasons why colo important or why bowel prep is needed; sometimes can suggest ways to do bowel prep (techniques like chill it) to make it better for ppts to do

Listen to ppt’s voice/response even when resistant as there is opportunity to ask questions and educate even if declining eventually

Ppt really likes post colo check in/ they often call or text PN, but also like the outreach to check in afterwards and often express gratitude or appreciation for help or service of PN

*Barriers or pain points*:

Reaching patients is hard, some patients decline and just don’t’ want it

Fear comes up in first call most often/ just need to understand the “whole process”

Barriers re transportation and escort more common, as are struggles with work schedule for booking it/ plus cost and insurance issues too

Pt’s don’t read prep instructions until day off so misunderstand the process/foods because start too late/ PN helps ppt’s avoid this if possible by verbally explaining when check to see got prep prescription/ also those with language barriers have harder time, need more support

Hard refusals, may hang up; or hard refusals due to prep even when PN explains reasons

Process of helping ppt’s get an application for financial assistance is timing consuming and with a lot of phone tag between organization, patient, and clinic – pt has to fill out app and have provider sign off which is a burden on ppt and programs often don’t try very hard to reach ppt’s who are challenging to reach

*Suggestions*:

Try to combined topics 4/5/6 somewhat depending on ppt and timing – pts often resting so reach out day before to check in or text them, often don’t call day of as ppt is resting, so may text or leave message in few days to say will check in on results in few weeks

Lessen outreach attempts as feels like overstepping at times with ppts who are not responding even after VM’s etc

Keep training and QA if sustain program and also initial outreach letter, all very helpful / improve tracking so have reminders of upcoming calls and topics, lessen the # of ppts by adding more navigators, keep outreach letter and make it electronic as well (my chart option)

*********************************************************

**Sampling of Extracted Quotes - deidentified**

**General/schedule and timing:**

*I think it's going good. It's getting a little…I mean, my patient load is getting a little bit more. So, but it was nice to have practiced with the pilot now that we're launch this. It's still…I hear pretty much a little bit of what I was expecting. A lot of people really appreciate this as a program. And then I do have a few that like rather not do it…would rather do it on their own or not have any assistance or anything. But the main challenge is just getting a hold of the patients was pretty much in the beginning and getting them scheduled. After that first relationship that built around the first call, then everything pretty much goes very smooth.*

**Helpfulness/ppt reaction**

*I know that they’re just very, very, very thankful throughout like the calls that I’m making with them. And I do see them get more comfortable with calling me even about little things. I tell them, hey, I have my cell phone with me at all times. Even if it’s something little and you’re not sure about it, just text me and I’ll be happy to answer. And even if it’s like a matter of them calling the clinic, they kind of ask me like, hey, is there something that you can look up or do I need to call my doctor? And just having that communication with them, that’s opened up a lot with most patients. I can’t think of a patient that’s not been like very appreciative about the program. Everyone is usually… Even if it’s like a reminder call where I’m not following up with the patients so much, or in so much detail, they’ll just be like, oh, that’s right. Yes. Thanks for calling me. You know, like I have everything good to go. It’s nice of you that you had that in your calendar and reminded me*.

**Advice, need for back up person:**

*Well, [Name] had mentioned before that I would have like [Name] as a backup. And eventually if this was some of the things, which I think I'm still managing right now. But, I'm kind of thinking like how soon will I need that help. And I'm trying to make sure that she understands everything. But I know that she also has her position. But it would be nice to have someone, like at least for the beginning and sit in the end of the week when it gets a little bit more saturated, when I'm getting a new batch of patients and having to review them. And at the same time at the end having to close those and like uploading and sending all these files in the meetings. That's usually where I struggle, at the beginning and at the end of the week. So like in-between I kind of catch up on reaching out to the patients. But when I do have patients scheduled, for example Monday or Friday, I kind of push them towards the middle of the week. And I…I mean, it works fine, but for example if I have someone that has…I have to check in before the colonoscopy, I don't want to, you know, push it out so that I’m checking after or…yeah, just those more time sensitive cases that I need to make sure I reach out to….*

**Documentation/time/scheduling**

*I don't have a preference on when I do really reach out to patients or doing the other part. The thing is, is just documenting. First reaching out to the patient I would…Once I have them on the phone I have to report on the EA chart, on the medical record. And then on REDCap. And then on my notes. And then, sometimes on the Excel sheet. So depending on what the response is. So, that can take up some time.*

**GI and scheduling challenges**

*And because even if I tried they won't schedule with me. I mean, they won't let me schedule the appointment for the patient. I can have them on the line, which I do offer that. But usually they'll be like, oh no, I'll call…I'll just call and then call you back. Like there's no need to do a three-way. And, or sometimes they're just waiting for some paperwork. Which when they're…when it's that case then I can give them the number if they don't have it. Or, any information that I have on my side.*

**Facilitators to Reach**

*So, again for the first call, I try to do…Like if I've never spoke to the patient yet then I try to do like morning, afternoon, evening like the protocol. And different days. I'll try a weekend here and there, but I usually try to do it between the weeks cause some people don't like to get their…you know, like they don't even know who's calling and then they're calling on the weekend. So, and usually it's a Saturday that I call. Not a Sunday. But I try to get those done within the week. I do try after hours, but like the latest I think is 6:00 or 7:00. …I have reached the patients and they know…they're expecting a call, even if they don't have a specific time then I do kind of play with the time a little bit more. Like I'll try any other time during the weekend. And then I know that they have my number or they've seen my number, so if they would still answer. And then during the week same. I just try to look back when I called last so that I'm calling at a totally different time.*

**Text facilitator**

*And depending on the topic. Because it's just like, I'll check in with you the day of the colonoscopy. Just, you know, they're fine with a text message. But even when I send them a text message sometimes they'll call me right away and just say like, oh, I got your message. Everything's going well. Which that works. But, I have other patients that have said, oh yeah, just send me a message. I'll probably be sleeping after the colonoscopy, or something*.

*And even through text messaging, I just send them a text like, hey, I know your colonoscopy is coming up. I want to make sure you have your bowel prep. Or let me know if you have any questions. And then it will just be as simple, oh, I got everything. Thanks. And it’s not like a big response that I’m expecting from them or anything.*

**FQHC Coordination**

*I would just say, if this was to take place with FQHC whether it’s with colonoscopies or anything else…I mean it’s been really helpful. But there’s so much things that are just not followed up with that I’ve found working with this program. So it’s nice to have like a few people working on the same patients. So I don’t know if they’d be like… Like, care coordinators here like we have three or four for all the cancers. And just knowing that there’s more than one person looking into their chart because I’ve found that some of the patients I’ve worked with have been getting good follow up with the medical assistants and the provider on getting this process done, even just getting enrolled with the community service, like BCCHP, for their colonoscopy. And there’s just kind of those… I mean closing those little gaps that, you know, someone left… I had a patient that filled out everything for Project Access. And then when I called him, he said, well, nobody has called me back. It’s been awhile. And I don’t know who called. And so I called the clinic. And it was more like, oh, the patient I was working…or the care coordinator that was working on his stuff is now gone. So everything is on hold. And we’re putting patient care on hold because somebody left? That’s just not okay. So just those little things that I don’t know if anybody would have noticed it if the patient doesn’t call. You know, I happened to call the patient because of this. But it’s just kind of scary in a way that so many things are left not totally done.*

**Delivering navigation/ppt reaction: refusals and or why join and follow thru**

*I have noticed that a lot of the times when patients are kind of like undecided about getting their colonoscopy done it's because they're just not very well informed. So, I like to ask them, you know, if explaining the procedure or the reason why it's needed like could help them. And then pretty much once I've done the explanation then they kind of make the decision on their own. And they realize why it's important. You know, I give them examples from the motivation interviewing and stuff that I have. But it's usually like at the beginning. And they're just like, oh no, I'm not going to do it. Like I don’t care for you to explain or anything. Or when I hear in their voice like, ‘oh, I probably will later’, then that's when I feel like I have more of a leeway to ask them more questions because they're unsure and then they're pretty open to listening or talking about it. But I do also get those hard refusals where they're like, ‘ no, I know what you're calling about and I've talked to my doctor about it. Like I don't…I don't care to do it’, so those are just like, okay, I can't argue. It's your decision.*

**Hard refusal example and how to motivate**

*The only one that I can think of that was pretty like, kind of like a hard refusal at the beginning, was because of the bowel prep. But I did explain…I mean, she still let me talk and explain to her that there was different types of bowel prep. And maybe she could discuss with her doctor. But I also explained how necessary it was for the prep to be done and like completely, because her problem was she would like vomit the prep and stuff like that. And pretty much just other things that have worked for other people. It's like drinking the liquid. Drinking it while it's pretty chill and…or using a straw or stuff like that. But she was kind of open to listening, once I got into that subject. But there's others that don't even let me ask. Like, no, no, and then they'll hang up so…[chuckles].*

*For the most part, they just tell me like, oh, I know how this works. Or either I have family that just did one. I had a lady that said, oh, my husband just did one and I helped him with it. So I know what I’m doing kind of thing. So I just do the check in.*

**Fear, work and transportation challenges and prep/fac and barriers**

*Or working on transportation. That's been a challenge for other patients. But usually, we figure out a way of either they end up finding someone else in the family or just a friend they trust that could take them. But it's kind of difficult at the beginning because the GI specialty will not schedule them unless they their companion. So, I try to make sure that they just find someone right away. Or if they have someone in mind, to schedule the appointment and then if…when it gets closer to the appoint in time, then make sure if we need to move it out then we can move it until we like have transportation. But, so that's one of them. The other one is fear. But that usually comes up right at the beginning. And then it's either fear of like the procedure itself, or I had a patient that was afraid of needles. Then it's like, yeah. So with the prep it's a little bit easier to explain because a lot of patients think that they have to be in this like total strict diet and they can't eat anything. And that was one of the things that patients were told, you know, what they have to avoid. But then they were left without knowing what was okay to eat. So, I've used other like clinic's instructions. I think it's Skagit that has like the food you can eat so that they at least have like an idea of, you know, they're not totally avoiding every…you know, everything. [Chuckles] I had one patient that was trying not to eat anything the whole day. And I was like, no, no, you can still have, you know, clear liquids and stuff like that. So just kind of clarification on that part. Even on the low fiber diet.*

*I think it’s a little bit still more of that companion thing…companion because like I do offer the list right away, the list services. And they’re like happy to hear. But I’ll still mention you still need a companion. So if you can get someone to go with you, I’ll schedule the ride. But it kind of gives them a little bit of a piece of mind like, okay. Even though they think like, oh, well if I have someone then they’ll give me a ride. I don’t need the ride. But it’s still kind of… I can hear it in their voice as like, okay, well if anything happens then I can just have my neighbor be with me. And I’ll have you schedule it.*

**Prep facilitators**

*On the clear liquid side, well, drinking the prep, I mean during the day, hey are pretty clear. But if they have any questions they usually just call me or text me like just to double check on something if it's not listed on their instructions. I feel like it [patient reach out during prep] does happen pretty often. So, not so much for like the days before on the low fiber diet. Once I give them the information it's pretty okay and understood. They do like double check like the types of broth or if they're making something at home, like the homemade broth. And then the juices that they drink. But yeah, they usually either, they'll address that the day before when I call them, or they'll randomly text me or call me when they're about to drink something or getting ready buy something. So yeah, it does happen.*

**Referral issues between clinic and GI/ schedule delays/ timing**

*Most of the delay is either they're still processing the referral or they never got it, which there's a way that I can pull that up and refax it so then check again in two days, cause that's kind of like for most their timeframe. About forty-eight hours, yeah. And then checking back with the patient because the patient would be the one that has to schedule. So that's kind of the backup voice thing. And that' on the specialty side it's not too difficult. It's more either kind of on our end whoever answers the referral and if it just didn't go through. And then I have some referrals on our side that are answered but there's no referral information. So, just messaging the provider…the MA. Either it just didn't go through or…Because they're like, ‘oh, I'm missing just the abnormal FIT or something’. Usually it's the whole referral that I---Like either it never was faxed or it was…I don't know… …I don't see the MA for anything so I don’t know how their process is. I'm pretty sure it's manually faxing, but I was thinking I don't know if electronically there's any issues there. But, yeah, cause I just find out when I call, or when the patient calls and they tell me.*

*Sometimes that’s the only thing that I see that once I get the patient on the phone for the initial call, whether I’ve tried a few times or if it’s the first time I’m calling, they have a quick turnaround from when they get the referral to scheduling. And they’re either in the process already of getting their colonoscopy. Pretty much, like they’re already doing their fiber diet or something like that within that week. So I kind of like just jump up to those steps. And I also have reached patients that had already completed it. And I’m like, wait. How did this happen? So those patients that I have that will take a long time to get scheduled. But I have found a few that it’s my initial call and they’ve already done it. And they already had their results, so…Because sometimes I get the data pool is the patients that have the abnormal FIT. And I start scrubbing. And then within that same week, I send out the letter. So I don’t call them until the following week which it gives them time to receive the letter. And I feel like it works out fine for most patients. But there’s some providers that they probably just put in the order, the referral order right away. And then the GI clinic has an opening so they get them scheduled right away. But there’s others that, even after I sent the letter, I’m making my initial call and I still don’t have the information on where they’re being referred. Like, I just know that they need to be referred. And it takes me longer time to contact the MA. And then the MA has a provider and does a referral.*

**Poor GI communication re preconsult vs colo**

*So whether they need it or not, like usually they’re told. The only thing is that I've had patients that are confused and they're thinking that they're going for their colonoscopy. And then they're worried because they don't have instructions, which most likely it's that it's been a pre-consult but it's not very clear what they scheduled. So I've learned to double check with them. As soon as they give me a date for their appointment I…Even before I ask, okay, when they schedule you make sure that you check if this is a consultation or if it's the actual procedure. And I think I've only had one patient, ‘why do I have to have a consultation?’ And I'm like, ‘well, it depends on the clinic’. But I think that they arranged it so that she had it like a Monday she had the pre- consultation and Friday she had the colonoscopy. And it was really a short timeframe, but it worked out for her so that…that turned out fine.*

**GI interaction**

*It’s been fine. I mainly have contact to them to like see if their schedule is open or just to find out why the patient hasn’t been scheduled. Sometimes it’s just a matter of refaxing the referral. But even when it’s come up where I’m trying to figure rides for patients and I’ve asked the schedulers, they’re pretty good at like referring me to like other resources that they use around their area. So I’ve been taking note of those.*

**Training/ QA/ challenges and benefits of multiple areas to document in**

*I mean, the trainings were really helpful. I really like that I was able to visit the GI clinic, even with patients. Because it was more of like hearing what their barriers were. But also seeing for myself things that would come up that they probably wouldn't have mentioned. And…I mean, the documenting has been really helpful. Having it in different things is not like the best. [Chuckles] But because I have to document everywhere and it's just like taking more time. But I think it's still been really helpful like if something goes missing I'm able to refer somewhere else and…[Regarding QA/pilot work}: Yeah. Well, yeah, especially at the beginning when I was a little bit more worried about like where it's really, really strict with verbatim scripting. Or, do I kind of, you know, let it be flexible a little bit, depending on the patient. And just kind of keeping the balance, like I didn't want to go really off the grid either. But she would…It seems that I would leave out especially like at the beginning of the call, like is now a good time to talk to you, or…because I was so determined to get the patient, you know, familiar with the study and everything I would forget to ask little things like that. So, after [QA} would tell me, oh, little things, like little comments like that. Like it would be nice if you asked. Because then I would find out during the call like, oh, that probably wasn't the best time cause I could hear kids crying in the background or something. [Chuckles] So yeah. Or other comments that she's made that's been helpful. And usually it's like, yeah, I was thinking the same thing, but it's nice to hear it from her. And like, kind of like getting some confirmation in a way. Then when I'm kind of iffy about what I should say. I think it makes it a little bit not so natural when I know that she's listening. [Chuckles] But at the same time it helps. But it helps knowing that…her comments and stuff that I might not notice at the time of the call…before the QA policy of just practicing, we did like for ten patients. And [Name] would be like a difficult patient, or sometimes she would be like just someone that was fine with it, going through with it. So it was nice to get that feel for what I might expect. Yeah. And then, I guess just the check-ins with either with like her or [Name] whenever I have check-ins. It's like, if I have something I try to save it for that time. But even when I have something right away that needs to be addressed, they're pretty quick answering my questions.*

**Training/documentation**

*I feel like documentation. And that’s, I think, for anything. I’ve been telling her even if you think it’s like a silly note like, oh, I called but they didn’t answer, like that’s already letting me know if you were gone or if someone is picking up your work, the patient wasn’t totally ignored. You called. They didn’t answer. Or even if it’s repetitive, called, called, called, we know that something is being done and it’s not just being ignored. So I think I’m stressing that out more with her…When I was gone and she covered me on my vacation, she did pretty good notes. But just because everything with the EHR that we have currently is still… I mean we’re now with EPIC. But the older one, there’s so many places that you can document things. So I’m just like, if you can keep everything noted in one spot and make sure it reflects, you know, the red cap, for example, that you’re not able to see a lot of patient information but just consolidate those things so that you’re not doing the work over or having to like review extra charts just because you’re not sure if things were done or where you left off.*

**Patients find most helpful**

*I think it's mainly around the education part. So, I…Not just at the beginning but just explaining like, again why it's important or why they're doing it. Kind of reassuring them. But I've noticed that some patients, even though I know that it sounds like they're going through with the colonoscopy, they kind of just like to double check things just to be sure. So, they like to call me instead of calling the clinic, or they'll tell me like, oh, do you mind calling the clinic and double checking this for me? I forgot to ask this. And I know that they can probably do it, you know, on their own. But there's still patients that kind of want that like, will you make sure for me since you know what you're talking about, or just stuff like that.*

**Post colo results call and pt appreciation**

*They're just very thankful. They're like,’ oh, everything went so well. I'm so glad that I did it because they found this’. So, then it's more like I listen to them on that part. And kind of, you know, deemphasize why it was so important. And also when they have to be on surveillance for it. So if they have to repeat it in three years or five years like I can hear in their voice that like they're more likely to do it. They were just like, I've done this before, like I can do it….And then even also when I've had patients that they had like three [cancers?- polyps?], they've only had one. That they were like afraid but then they're still like so thankful and they're just waiting for like the pathology report. But they kind of appreciate that double checking.*

**Decline reasons or use of text to limit calling someone who needs less help**

*I've had a patient that pretty much told me they're not interested in like the navigation part of it. But just because they've done it that they find that less helpful. It was more like pretty much I know that people would love that you help them but I can do it on my own. [Chuckles] And then I've offered, ‘okay, then is, you know, a check-in okay? You know, so you have my number and let me know if you have any questions’. But like that's been like, ‘oh no, that's okay. Don't worry about even contacting me’. So, it's either been that or if anything is where I have mentioned like, oh, I'll be checking in with you several times then I'll…at the end of each call tell them when I will check in again. If they're pretty familiar with the colonoscopy, or they don't want so much like the check-ins I always offer the text messages. It's like, oh yeah, you don't have to call me. Just text me. And I'll let you know if…you know, if I have like questions, and yeah.*

**Transportation/escort issues**

*And that's the trouble with one of our patients. Like they'll find a way to get there but they need someone to be there. So it's more like having that caregiver or that person that's there. But I mean, I've been able to go with patients that are here in the area. I went with another lady. And it turned out that her ride had cancelled like kind of last minute so I did give her a ride. So that worked out. I mean, and I was there for during the appointment so that was fine. And then it's just more of those that are long distance that I, you know, trying to figure out a way. But it's gotten to the point that patients are still like looking, you know, if a family member can't do it, a close friend or just another relative, or…And it just takes time and it gets closer to the appointment time, but they end up finding someone*.

**Reach, topic, timing barriers**

*When I have patients that won't answer and like I'll have them enrolled in the study and like I'm ready to do the check-in, but then I don't get anymore answers. And so I know I'm supposed to do like the sixteen calls for each topic and I feel like it can be a lot like maybe one of these days they're going to answer and they're going to be so mad. [Chuckles] Because I still have to go through each topic.*

*It takes maybe the first two or three calls with no answer, leaving a voice message and even…yeah, jus leaving a message. The letters help out a lot. I feel like I don’t get response from everyone, so…that goes the week before. So some people will call me, you know, like the next week when I’m supposed to start making the calls. They either call me before or they’re already looking out for my call.*

*I know we’re also doing the text messaging. That’s new. So we’ll see how that… I would think it would give it like an extra push to get patients to either answer or call us back. I mean I remember more, to make a follow up call, when I get a text message from like my dentist. So like once I see it, I’m able to do something or at least schedule it in my calendar. Because they call me and I forget to even listen to the voice message. And so it’s like flashing on my phone or something. So I feel like we’re more…I mean I don’t know about… It depends on the patients. But I’m more open to like looking at my messages between the day than like waiting until my day is over and I can make the call*.

**Advice/combining of topics or # of call attempts**

*It's the day of the before the colonoscopy and then…Some of those I'm able to combine and not like the whole topic, but answer part of the questions. For example, for the day…I think…Oh, the day of the colonoscopy I don't really call until after because most of the time they're resting. So I'll either touch base on some of the things the day before, or I'll call the day after. Or I'll just send them a text and then I'll tell them I'll text in two weeks with you when we have the results. Because at that point they usually don't have too much information except for, you know, the procedure report. That they have already been told that they have to wait two weeks to get like pathology results.*

*So, we do eighteen calls. I would do…I mean I would still do like the six calls, trying to get six calls within a week and then… Because we’re already sending like the letter the week before then we do six calls. Within that week, I would do… Now, that we’re doing the text message, do the text message and then some calls the next week. And then I would just like stop there because I feel like they have enough information to be able to contact us back, whether it’s like text message or giving us a call, voicemail or anything, if they’re really needing any type of assistance. And I try to be very specific, in the voicemail, without leaving too much personal information for the patient. That I’m here to help with the referral process. I’m here to, you know, make things easier for them. And I can tell that right away they really want to know. Even if they’re confused about what referral or they didn’t even know that they had a referral, they’ll call if they’re willing to do something. But I feel like sometimes it crosses the line. And am I just being annoying to the patient now because I know that he’s ignoring my calls. Or you know, like when I call and you can tell that it was sent to voicemail, like purposely. It’s like, okay, do I keep calling?*

**Additional staffing support/ helps with reach and workload**

*I think this works fine. Or just having like the extra person to…even if we’re like doing the work together, like making the calls, I could be doing something while she’s making the call. If they answer, then she’ll transfer. I’ll stop doing whatever I’m doing. And then you know, like I’ll take that call. Or if she just needs to document and leave a message then she’ll do that. And I know that it’s still being worked on. And I can work on stuff that I don’t need to be on the phone for. So that’s been working out good. And I think also I was trying to find the balance between getting all those calls done within that week and, at the same time, making sure that I’m not ignoring the calls that I already have scheduled on the calendar for people that already started navigation. So between trying to get people to start navigated…being navigated and then the ones that are already in the process.*
